# Supplementary figures and images for: Identification of pathways to high-level vancomycin resistance in Clostridioides difficile that incur high fitness costs in key pathogenicity traits
Source: PLoS Biol. 2024 Aug 15;22(8):e3002741. doi: 10.1371/journal.pbio.3002741 (PMC11326576; doi:10.1371/journal.pbio.3002741)

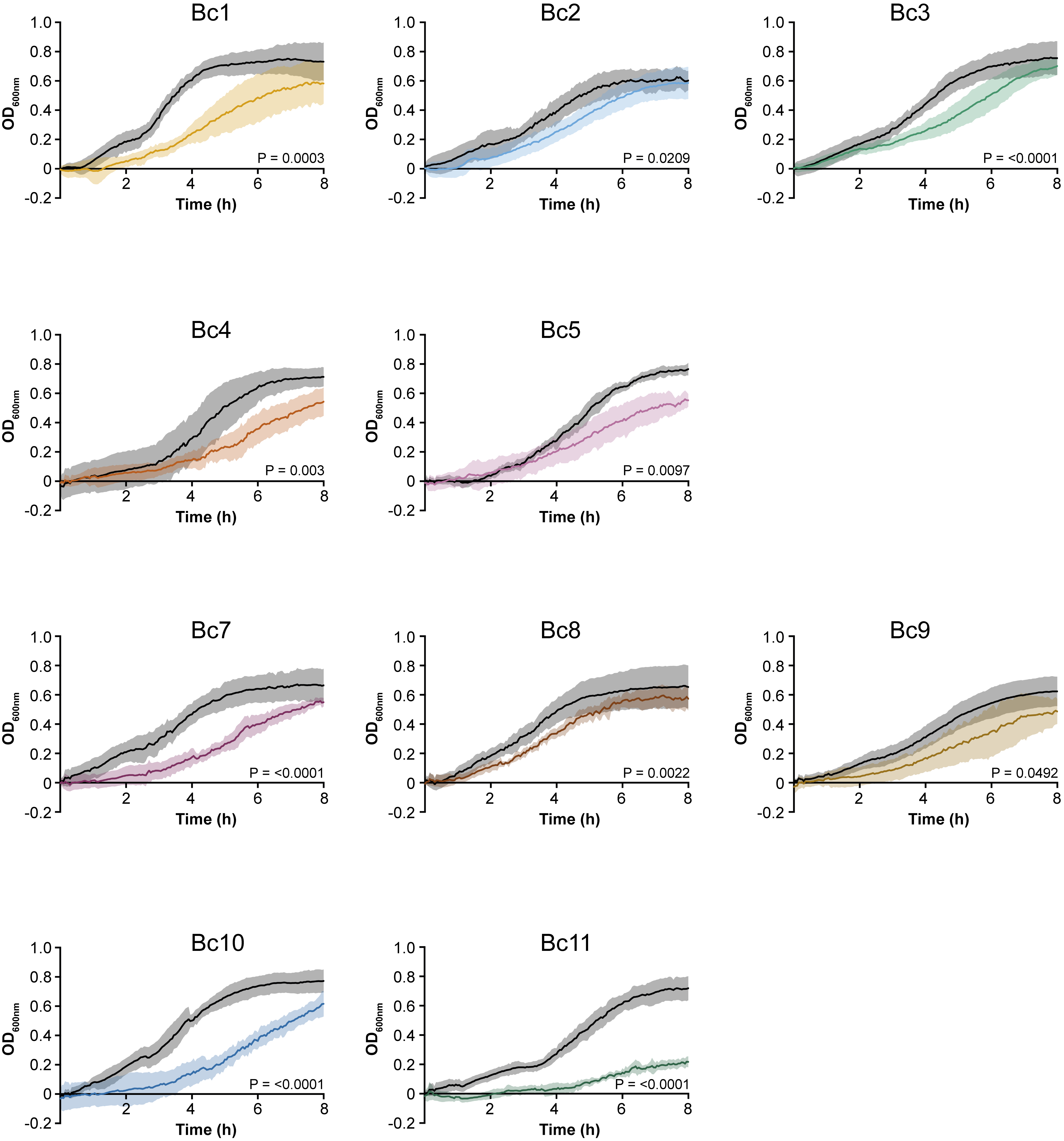

Supplement: S1 Fig — Growth over time in rich media (TY broth) was measured at 600 nm in a 96-well microplate spectrometer. Growth of each endpoint clone (coloured lines) was compared to its matched control (black lines). Shown are the mean and standard deviation of repeats, assayed at minimum in biological and technical triplicate. For each strain, area under the curve was determined using the GrowthCurver R package and these were compared using Student’s t tests with Welch’s correction, with the P-value shown on each graph. All pairwise differences are significant. The data underlying this figure can be found in S5 Data. (TIF) [file pbio.3002741.s001.tif]

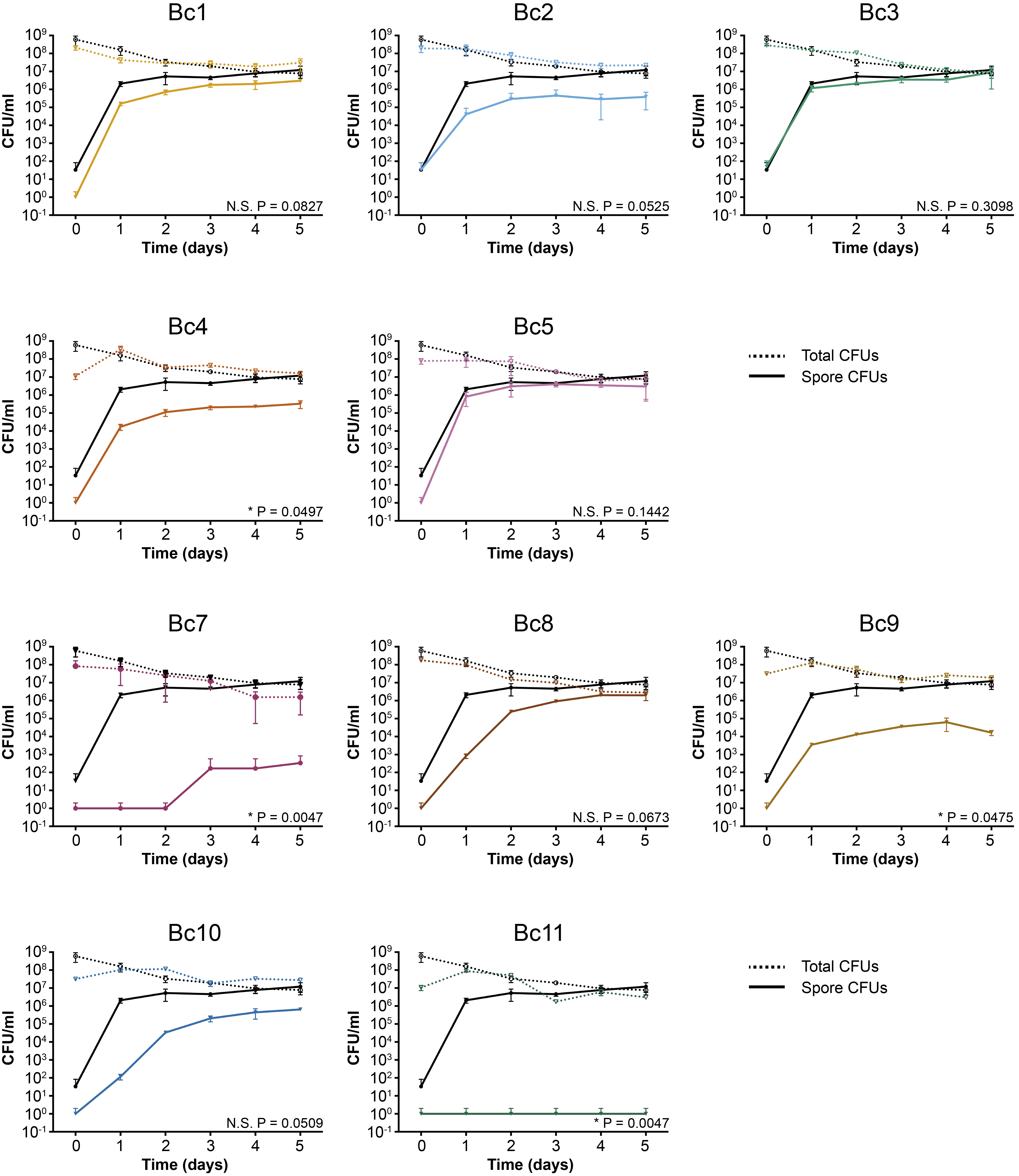

Supplement: S2 Fig — Sporulation efficiencies of each endpoint clone (coloured lines) were compared to the parental R20291ΔPaLoc (black lines). Stationary phase cultures were incubated anaerobically for 5 days with samples taken daily to enumerate total colony-forming units (CFUs, dotted lines) and spores (solid lines), following incubation at 65°C for 30 min to kill vegetative cells. Shown are the mean and standard deviations of biological duplicates assayed in triplicate. For each strain, spore CFU area under the curve was determined using Graphpad Prism and these were compared using Dunnett’s T3 multiple comparisons test with the adjusted P-value shown on each graph. * = significant difference, N.S. = not significant. The data underlying this figure can be found in S5 Data. (TIF) [file pbio.3002741.s002.tif]

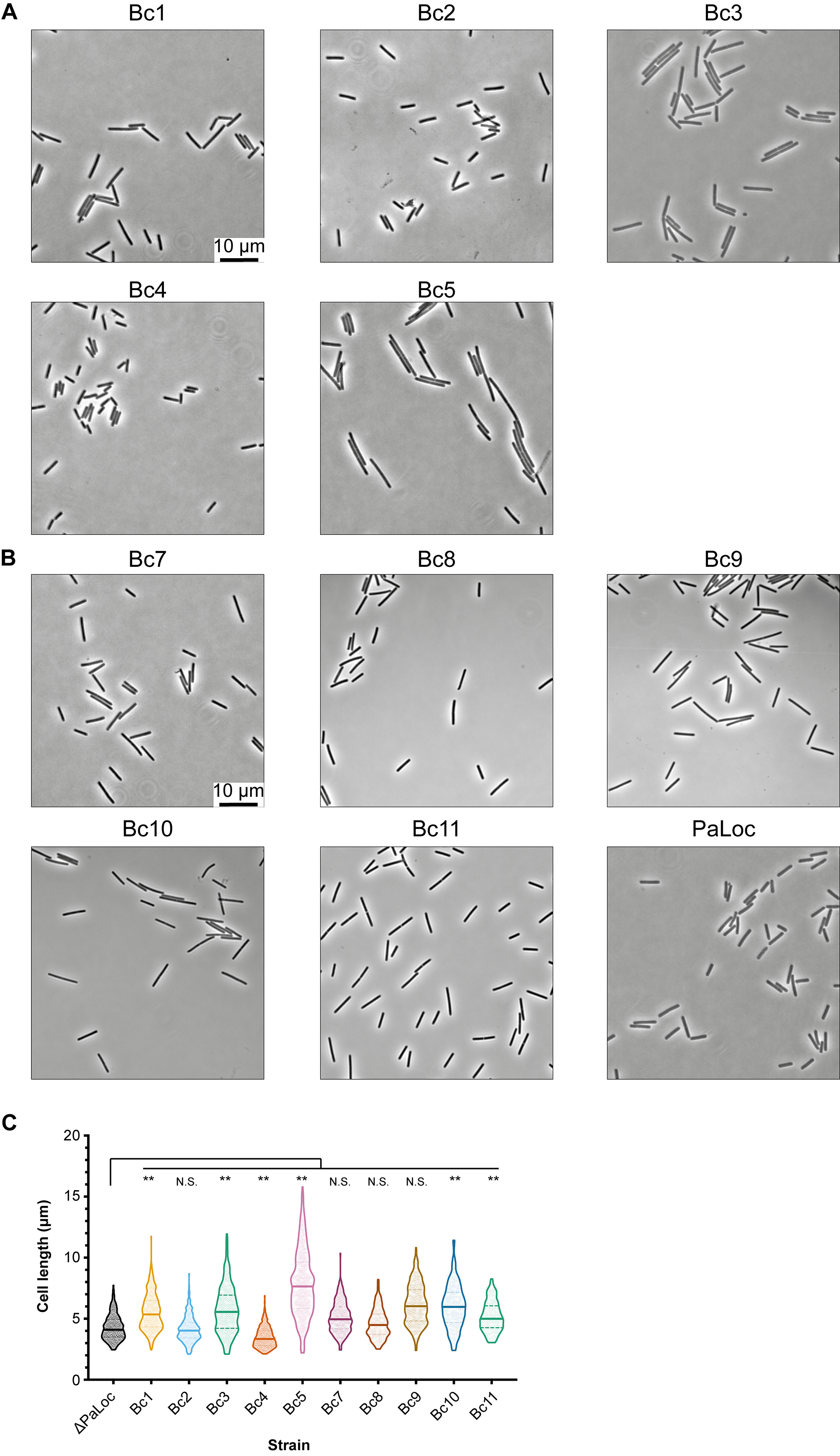

Supplement: S3 Fig — Phase contrast light microscopy of mid-log cultures of each wild-type (A) and hyper-mutating (B) endpoint clone, with R20291ΔPaLoc for comparison. Shown is a representative field of view for each strain. (C) Imaging was performed on biological triplicate cultures and images were analysed using MicrobeJ to determine lengths of at least 185 individual cells for each strain. Shown is an all point violin plot with the median indicated by a solid horizontal line. Statistical significance of evolved isolates against the R20291ΔPaLoc control was calculated using a one-way ANOVA with Dunnett’s T3 multiple comparisons test, ** = P < 0.0001, N.S. = not significant. The data underlying panel C can be found in S5 Data. (TIF) [file pbio.3002741.s003.tif]

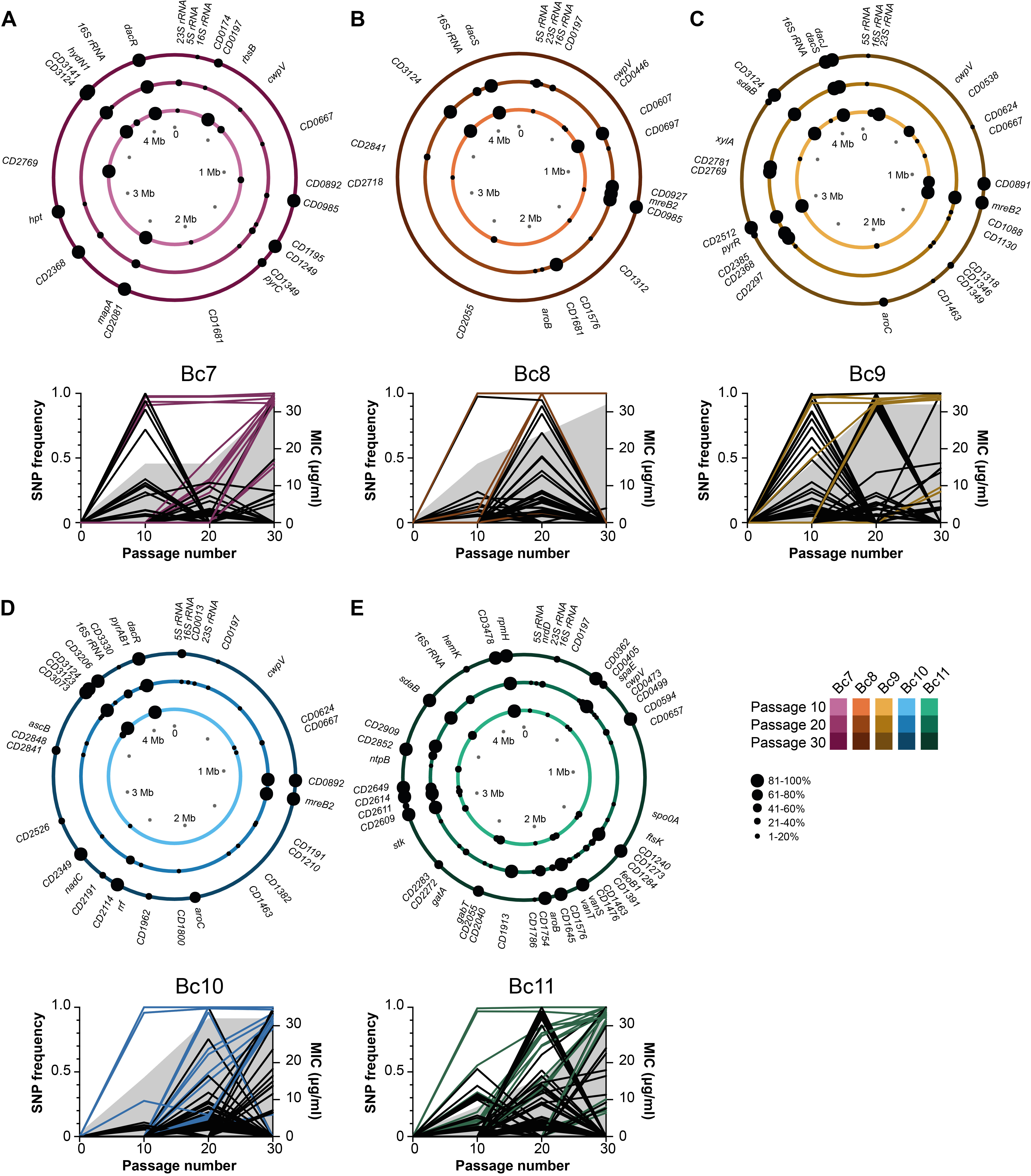

Supplement: S4 Fig — Accumulation of variants in the hyper-mutating C. difficile lineages Bc7 (A), Bc8 (B), Bc9 (C), Bc10 (D), and Bc11 (E). Each circle plot represents the 4.2 Mb genome of a single evolving population after 10 (inner ring), 20 (middle ring), and 30 passages (outer ring), with the locations of non-synonymous within gene variants indicated with black circles and the penetrance of each mutation in the population indicated by the size of the circle. The line graphs show the frequency of all variants (intergenic, synonymous, non-synonymous, and nonsense) in each population. The vancomycin MIC for each population is also indicated by the shaded region. Mutations also identified in the respective end point clone (Fig 1C) are highlighted by the coloured lines. The data underlying this figure, including a full list of all variants shown here, can be found in S3 Data. (TIF) [file pbio.3002741.s004.tif]

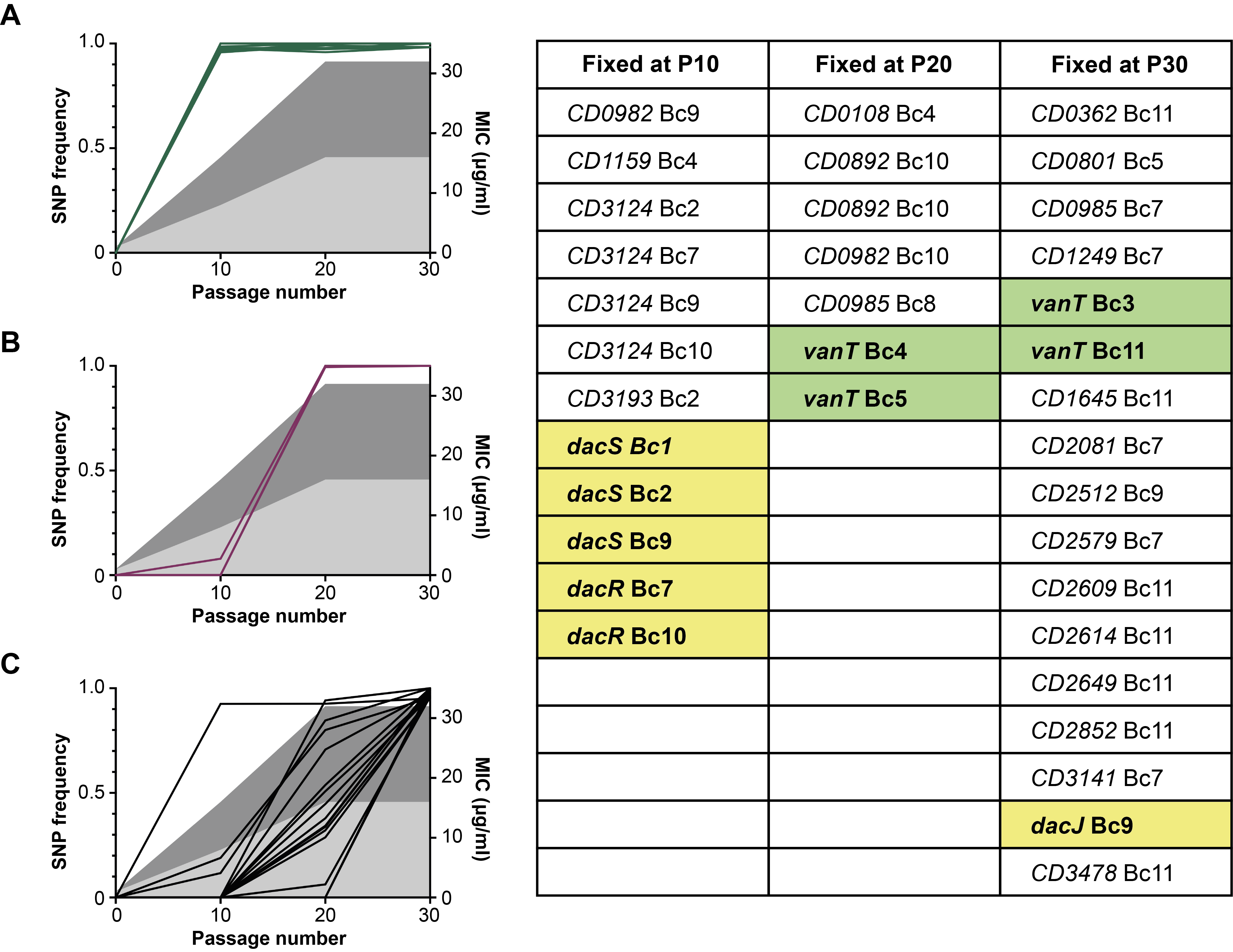

Supplement: S5 Fig — Shown are the individual variants which fix (>95% penetrance) in all 10 parallel populations during vancomycin resistance evolution after 10 (A), 20 (B), and 30 passages (C). The frequency of each variant within their respective population is shown and the genes affected at each time point are shown in the table on the right. The genes in the dacJRS cluster are highlighted in yellow and vanT (CD1526) in green. The range of vancomycin MICs observed across all populations (lowest, light grey; highest, dark grey) is indicated by the shaded areas. The data underlying this figure can be found in S3 Data. (TIF) [file pbio.3002741.s005.tif]

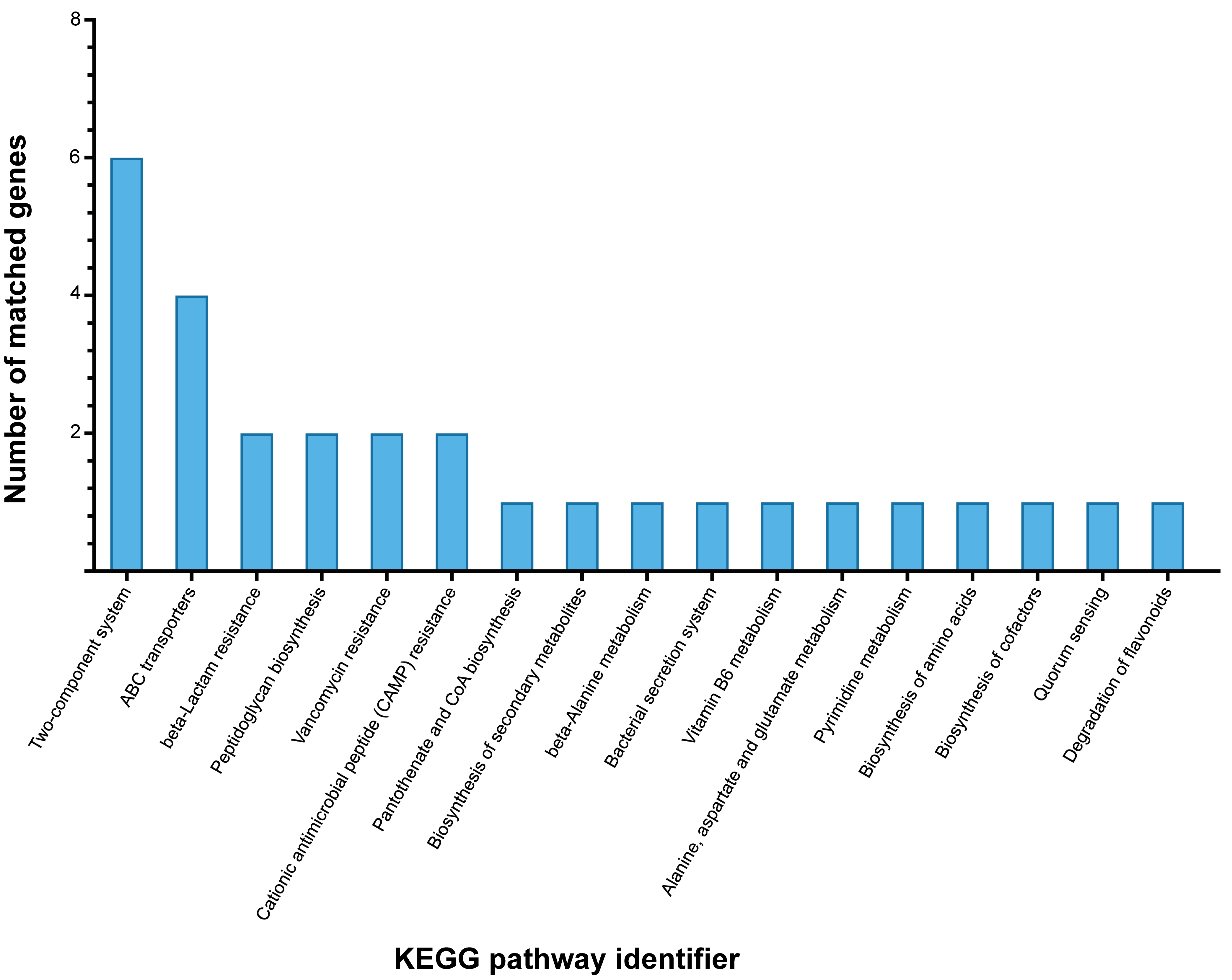

Supplement: S6 Fig — Genes impacted by mutations during evolution (except those in the transiently hyper-mutating Bc1 P20) were visualised in KEGG (Kyoto Encyclopedia of Genes and Genomes) colour mapper and assigned to cellular pathways. Two-component systems and ABC transporters were the best-represented functional classes. The data underlying this figure can be found in S5 Data. (TIF) [file pbio.3002741.s006.tif]

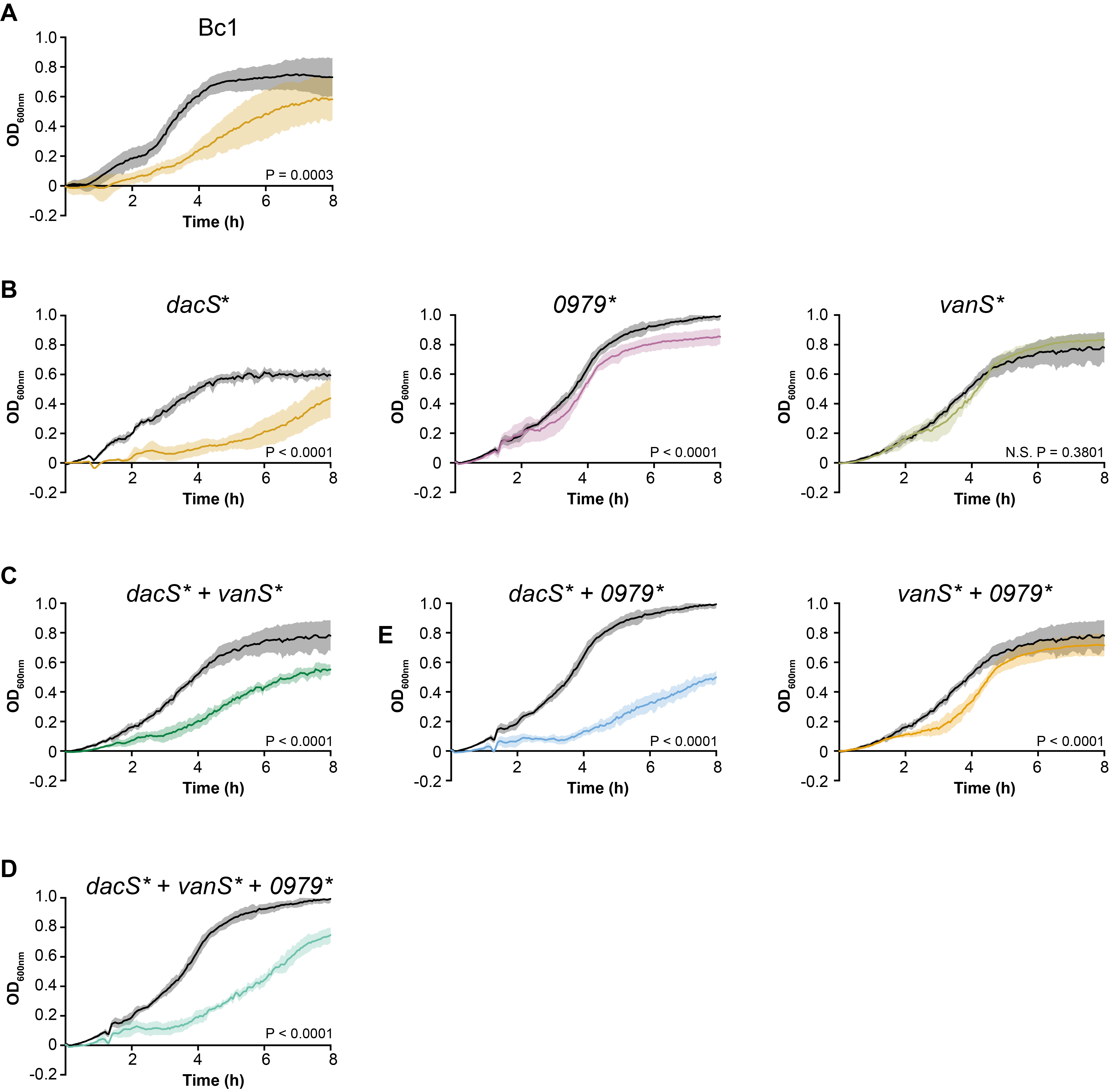

Supplement: S7 Fig — Growth over time in rich media (TY broth) was measured at 600 nm in a 96-well microplate spectrometer. Growth of endpoint clone Bc1, R20291ΔPaLoc dacSc.714G>T (dacS*), 1,197,357_1,197,400del (0979*), and vanSc.367_396dup (vanS*) single, double, and triple mutants (coloured lines) were compared to R20291ΔPaLoc (black lines). Shown are the mean and standard deviation of repeats, assayed at minimum in biological and technical triplicate. For each strain, area under the curve was determined using the GrowthCurver R package and these were compared using Student’s t tests with Welch’s correction, with the P-value shown on each graph. N.S. indicates differences were not significant. The data underlying this figure can be found in S5 Data. (TIF) [file pbio.3002741.s007.tif]

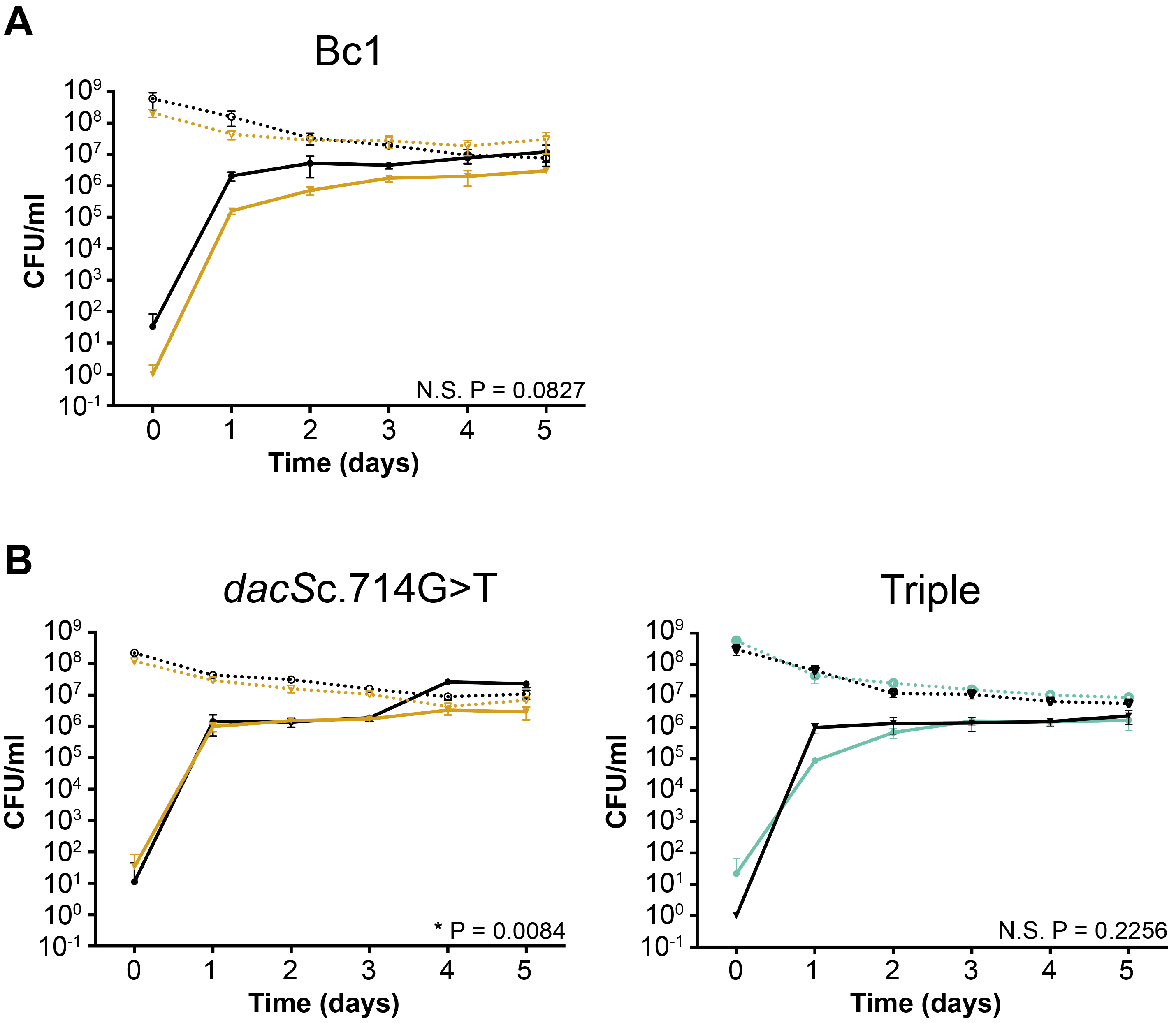

Supplement: S8 Fig — Sporulation efficiencies of endpoint clone Bc1, R20291ΔPaLoc dacSc.714G>T and the dacSc.714G>T 1,197,357_1,197,400del vanSc.367_396dup triple mutant (coloured lines) were compared to R20291ΔPaLoc (black lines). Stationary phase cultures were incubated anaerobically for 5 days with samples taken daily to enumerate total colony forming units (CFUs, dotted lines) and spores (solid lines), following incubation at 65°C for 30 min to kill vegetative cells. Shown are the mean and standard deviations of biological triplicates assayed in triplicate. For each strain, spore CFU area under the curve was determined using Graphpad Prism and these were compared using Dunnett’s T3 multiple comparisons test with the adjusted P-value shown on each graph. * = significant difference, N.S. = not significant. The data underlying this figure can be found in S5 Data. (TIF) [file pbio.3002741.s008.tif]

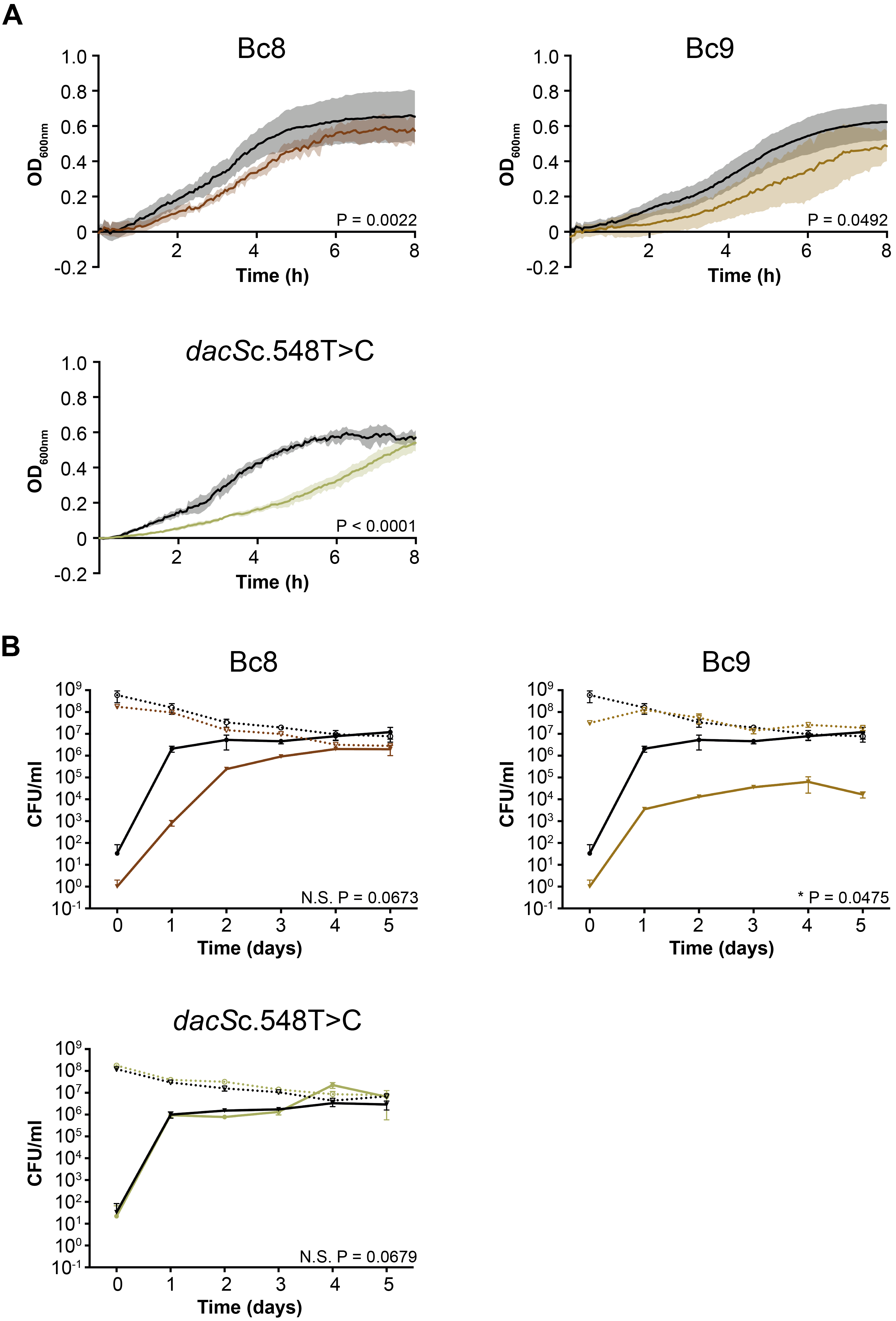

Supplement: S9 Fig — (A) Growth over time in rich media (TY broth) was measured at 600 nm in a 96-well microplate spectrometer. Growth of R20291ΔPaLoc dacSc.548T>C and endpoint clones Bc8 and Bc9 (coloured lines) were compared to matched controls (black lines). Shown are the mean and standard deviation of repeats, assayed at minimum in biological and technical triplicate. For each strain, area under the curve was determined using the GrowthCurver R package and these were compared using Student’s t tests with Welch’s correction, with the P-value shown on each graph. All pairwise differences were significant. (B) Sporulation efficiencies of R20291ΔPaLoc dacSc.548T>C and endpoint clones Bc8 and Bc9 (coloured lines) compared to the parental R20291ΔPaLoc (black lines). Stationary phase cultures were incubated anaerobically for 5 days with samples taken daily to enumerate total colony forming units (CFUs, dotted lines) and spores (solid lines), following incubation at 65°C for 30 min to kill vegetative cells. Shown are the mean and standard deviations of biological triplicate assayed in triplicate. For each strain, spore CFU area under the curve was determined using Graphpad Prism, and these were compared using Dunnett’s T3 multiple comparisons test with the adjusted P-value shown on each graph. * = significant difference, N.S. = not significant. The data underlying this figure can be found in S5 Data. (TIF) [file pbio.3002741.s009.tif]
