# Supplementary material for: Identification of pathways to high-level vancomycin resistance in Clostridioides difficile that incur high fitness costs in key pathogenicity traits
Source: PLoS Biol. 2024 Aug 15;22(8):e3002741. doi: 10.1371/journal.pbio.3002741 (PMC11326576; doi:10.1371/journal.pbio.3002741)
Supplement: S1 Table — (DOCX) [file pbio.3002741.s015.docx]

**S1 Table:** Strains used in this study

| **Strain** | **Characteristics** | **Source** |  |
| --- | --- | --- | --- |
| ***General Strains – C. difficile*** | | |  |
| R20291 | *C. difficile* ribotype 027 strain isolated during an outbreak at Stoke Mandeville hospital, UK in 2006. | [1] |  |
| R20291∆*PaLoc* | R20291 with the entire pathogenicity locus (*tcdD, tcdB, tcdE, tcdA, tcdC)*, except the first codon of *tcdC*, deleted. | This study | |
| R20291∆*PaLoc*Δ*mutSL* | R20291∆*PaLoc* with the entire *mutSL* locus (*mutS, mutL*), except the first codon of *mutS* and the last 2 codons of *mutL*, deleted. | This study | |
| ***General Strains – E. coli*** | | | |
| CA434 | *E. coli* conjugative donor. HB101 carrying R702. | [2] | |
| NEB5α | *fhuA2 Δ(argF-lacZ)U169 phoA glnV44 Φ80Δ (lacZ)M15 gyrA96 recA1 relA1 endA1 thi-1 hsdR17.* | New England Biolabs | |
| ***Barcoded Strains*** | | | |
| R20291∆*PaLoc pyrE::*barcode 1 | R20291∆*PaLoc* with a 218 bp insertion between *CD0188* (*pyrE*) and *CD0189,* including 9 bp Barcode 1 (AAGTCCTCG) | This study | |
| R20291∆*PaLoc pyrE::*barcode 2 | R20291∆*PaLoc* with a 218 bp insertion between *CD0188* (*pyrE*) and *CD0189,* including 9 bp Barcode 2 (TCTTGACCG) | This study | |
| R20291∆*PaLoc pyrE::*barcode 3 | R20291∆*PaLoc* with a 218 bp insertion between *CD0188* (*pyrE*) and *CD0189,* including 9 bp Barcode 3 (AACAACACC) | This study | |
| R20291∆*PaLoc pyrE::*barcode 4 | R20291∆*PaLoc* with a 218 bp insertion between *CD0188* (*pyrE*) and *CD0189,* including 9 bp Barcode 4 (AACAGGTGG) | This study | |
| R20291∆*PaLoc pyrE::*barcode 5 | R20291∆*PaLoc* with a 218 bp insertion between *CD0188* (*pyrE*) and *CD0189,* including 9 bp Barcode 5 (ACCGATTAG) | This study | |
| R20291∆*PaLoc*Δ*mutSL pyrE::*barcode 7 | R20291∆*PaLoc*Δ*mutSL* with a 218 bp insertion between *CD0188* (*pyrE*) and *CD0189,* including 9 bp Barcode 7 (CCTCCAACT) | This study | |
| R20291∆*PaLoc*Δ*mutSL pyrE::*barcode 8 | R20291∆*PaLoc*Δ*mutSL* with a 218 bp insertion between *CD0188* (*pyrE*) and *CD0189,* including 9 bp Barcode 8 (CGAGGACAT) | This study | |
| R20291∆*PaLoc*Δ*mutSL pyrE::*barcode 9 | R20291∆*PaLoc*Δ*mutSL* with a 218 bp insertion between *CD0188* (*pyrE*) and *CD0189,* including 9 bp Barcode 9 (CTGGTTCTA) | This study | |
| R20291∆*PaLoc*Δ*mutSL pyrE::*barcode 10 | R20291∆*PaLoc*Δ*mutSL* with a 218 bp insertion between *CD0188* (*pyrE*) and *CD0189,* including 9 bp Barcode 10 (GGATGTTGG) | This study | |
| R20291∆*PaLoc*Δ*mutSL pyrE::*barcode 11 | R20291∆*PaLoc*Δ*mutSL* with a 218 bp insertion between *CD0188* (*pyrE*) and *CD0189,* including 9 bp Barcode 11 (GTCACCAGT) | This study | |
| ***Evolved Strains*** | | | |
| Bc1 | R20291∆*PaLoc pyrE::*barcode 1 isolated after 60 days of vancomycin selection pressure. | This study | |
| Bc2 | R20291∆*PaLoc pyrE::*barcode 2 isolated after 60 days of vancomycin selection pressure. | This study | |
| Bc3 | R20291∆*PaLoc pyrE::*barcode 3 isolated after 60 days of vancomycin selection pressure. | This study | |
| Bc4 | R20291∆*PaLoc pyrE::*barcode 4 isolated after 60 days of vancomycin selection pressure. | This study | |
| Bc5 | R20291∆*PaLoc pyrE::*barcode 5 isolated after 60 days of vancomycin selection pressure. | This study | |
| Bc7 | R20291∆*PaLoc*Δ*mutSL pyrE::*barcode 7 isolated after 60 days of vancomycin selection pressure. | This study |  |
| Bc8 | R20291∆*PaLoc*Δ*mutSL pyrE::*barcode 8 isolated after 60 days of vancomycin selection pressure. | This study |  |
| Bc9 | R20291∆*PaLoc*Δ*mutSL pyrE::*barcode 9 isolated after 60 days of vancomycin selection pressure. | This study |  |
| Bc10 | R20291∆*PaLoc*Δ*mutSL pyrE::*barcode 10 isolated after 60 days of vancomycin selection pressure. | This study |  |
| Bc11 | R20291∆*PaLoc*Δ*mutSL pyrE::*barcode 11 isolated after 60 days of vancomycin selection pressure. | This study |  |
| ***Engineered Mutant Strains*** | | |  |
| Bc1∆*dacJ* | Evolved endpoint isolate Bc1 with the entire *dacJ* ORF, except for the first and last codon, deleted. | This study |  |
| R20291∆*PaLoc*∆*dacRS* | R20291∆*PaLoc* with the complete ORFs for *dacR* and *dacS* deleted. | This study |  |
| R20291∆*PaLoc dacS*c.548T>C | R20291∆*PaLoc* with *dacS* 548T>C point mutation identified in Evolved R20291∆*PaLoc*Δ*mutSL pyrE::*barcodes 8 and 9. | This study |  |
| R20291∆*PaLoc dacS*c.714G>T | R20291∆*PaLoc* with *dacS* 714G>T point mutation identified in Evolved R20291∆*PaLoc pyrE*::barcode 1. | This study |  |
| R20291∆*PaLoc dacS*c.714G>T *vanS*c*.*367_396dup | R20291∆*PaLoc* with the *dacS* 714G>T point mutation and the *vanS* 30 bp duplication identified in Evolved R20291∆*PaLoc pyrE*::barcode 1. | This study |  |
| R20291∆*PaLoc dacS*c.714G>T *vanS*c*.*367_396dup 1,197,357_1,197,400del | R20291∆*PaLoc* with the *dacS* 714G>T point mutation, the *vanS* 30 bp duplication and the intergenic 44 bp deletion identified in Evolved R20291∆*PaLoc pyrE*::barcode 1. | This study |  |
| R20291∆*PaLoc dacS*c.714G>T 1,197,357_1,197,400del | R20291∆*PaLoc* with the *dacS* 714G>T point mutation and the intergenic 44 bp deletion identified in Evolved R20291∆*PaLoc pyrE*::barcode 1. | This study |  |
| R20291∆*PaLoc vanS*c*.*367_396dup | R20291∆*PaLoc* with the internal *vanS* 30 bp duplication identified in Evolved R20291∆*PaLoc pyrE*::barcode 1. | This study |  |
| R20291∆*PaLoc vanS*c*.*367_396dup 1,197,357_1,197,400del | R20291∆*PaLoc* with the *vanS* 30 bp duplication and the intergenic 44 bp deletion identified in Evolved R20291∆*PaLoc pyrE*::barcode 1. | This study |  |
| R20291∆*PaLoc* 1,197,357_1,197,400del | R20291∆*PaLoc* with the intergenic 44 bp deletion identified in Evolved R20291∆*PaLoc pyrE*::barcode 1. | This study |  |

**References**

1. Stabler RA, He M, Dawson L, Martin M, Valiente E, Corton C, et al. Comparative genome and phenotypic analysis of *Clostridium difficile* 027 strains provides insight into the evolution of a hypervirulent bacterium. Genome Biol. 2009;10(9):R102. Epub 20090925. doi: 10.1186/gb-2009-10-9-r102. PubMed PMID: 19781061; PubMed Central PMCID: PMCPMC2768977.

2. Purdy D, O'Keeffe TA, Elmore M, Herbert M, McLeod A, Bokori-Brown M, et al. Conjugative transfer of clostridial shuttle vectors from *Escherichia coli* to *Clostridium difficile* through circumvention of the restriction barrier. Mol Microbiol. 2002;46(2):439-52. doi: 10.1046/j.1365-2958.2002.03134.x. PubMed PMID: 12406220.
