# Supplementary material for: Identification of pathways to high-level vancomycin resistance in Clostridioides difficile that incur high fitness costs in key pathogenicity traits
Source: PLoS Biol. 2024 Aug 15;22(8):e3002741. doi: 10.1371/journal.pbio.3002741 (PMC11326576; doi:10.1371/journal.pbio.3002741)
Supplement: S2 Table — (DOCX) [file pbio.3002741.s016.docx]

**S2 Table:** Primers used in this study

| Oligonucleotide | Sequence | Use |
| --- | --- | --- |
| ***Primers for Cloning*** | | |
| RF920 | CGTAGAAATACGGTGTTTTTTGTTACCCTATGGAATTTAGATATAAAAACCAATTC | Amplification of homology arm upstream of *PaLoc* with RF921 |
| RF921 | ATTTATTTTGGTGTGGACAACATTGGAATTAAATCAG | Amplification of homology arm upstream of *PaLoc* with RF920 |
| RF922 | AATTCCAATGTTGTCCACACCAAAATAAATGCC | Amplification of homology arm downstream of *PaLoc* with RF923 |
| RF923 | GGGATTTTGGTCATGAGATTATCAAAAAGGCCCAACTATGGAAAAACC | Amplification of homology arm downstream of *PaLoc* with RF922 |
| RF2066 | AATACGGTGTTTTTTGTTACCCTAGAGCTCCCACTTATAATTTCTAATGAAACTGTG | Amplification of homology arm upstream of *mutSL* with RF2067 |
| RF2067 | CCAAATATTTTACATCATTATCAAACCTCCTTCTTTTC | Amplification of homology arm upstream of *mutSL* with RF2066 |
| RF2068 | GGAGGTTTGATAATGATGTAAAATATTTGGATATTTAAAATATATGGAAAG | Amplification of homology arm downstream of *mutSL* with RF2069 |
| RF2069 | TTGGTCATGAGATTATCAAAAAGGGGATCCGCCCTTTAACTTGCACTC | Amplification of homology arm downstream of *mutSL* with RF2068 |
| ***Primers for Barcoding*** | | |
| RF1810 | GAAAAAGGCTTCTCTCATGAGAAG | To linearise pJAK081 to add barcode fragments |
| RF1811 | GGTACCATAAAAATAAGAAGCCTGC | To linearise pJAK081 to add barcode fragments |
| RF1902 | ACC GAAAAAGGCTTCTCTCATGAGAAG | Inverse PCR of pJAK201 to introduce barcode 3 |
| RF1903 | GTTGTT AAATGGAAGATGGAATAGAAGTAAGC | Inverse PCR of pJAK201 to introduce barcode 3 |
| RF1904 | GTGG GAAAAAGGCTTCTCTCATGAGAAG | Inverse PCR of pJAK201 to introduce barcode 4 |
| RF1905 | CTGTT AAATGGAAGATGGAATAGAAGTAAGC | Inverse PCR of pJAK201 to introduce barcode 4 |
| RF1906 | GATTAG GAAAAAGGCTTCTCTCATGAGAAG | Inverse PCR of pJAK201 to introduce barcode 5 |
| RF1907 | GGT AAATGGAAGATGGAATAGAAGTAAGC | Inverse PCR of pJAK201 to introduce barcode 5 |
| RF1912 | CAACT GAAAAAGGCTTCTCTCATGAGAAG | Inverse PCR of pJAK201 to introduce barcode 7 |
| RF1913 | GAGG AAATGGAAGATGGAATAGAAGTAAGC | Inverse PCR of pJAK201 to introduce barcode 7 |
| RF1914 | GACAT GAAAAAGGCTTCTCTCATGAGAAG | Inverse PCR of pJAK201 to introduce barcode 8 |
| RF1915 | CTCG AAATGGAAGATGGAATAGAAGTAAGC | Inverse PCR of pJAK201 to introduce barcode 8 |
| RF1916 | GTTCTA GAAAAAGGCTTCTCTCATGAGAAG | Inverse PCR of pJAK201 to introduce barcode 9 |
| RF1917 | CAG AAATGGAAGATGGAATAGAAGTAAGC | Inverse PCR of pJAK201 to introduce barcode 9 |
| RF1918 | TTGG GAAAAAGGCTTCTCTCATGAGAAG | Inverse PCR of pJAK201 to introduce barcode 10 |
| RF1919 | CATCC AAATGGAAGATGGAATAGAAGTAAGC | Inverse PCR of pJAK201 to introduce barcode 10 |
| RF1920 | CAGT GAAAAAGGCTTCTCTCATGAGAAG | Inverse PCR of pJAK201 to introduce barcode 11 |
| RF1921 | GTGAC AAATGGAAGATGGAATAGAAGTAAGC | Inverse PCR of pJAK201 to introduce barcode 11 |
| ***Primers for qPCR*** | | |
| RF2504 | CATCATTACCAGGTGTAGCAGTG | Amplification of ~200bp *rpoA* fragment for qPCR |
| RF2505 | GGAGGACAGATTATATCTGCACC | Amplification of ~200bp *rpoA* fragment for qPCR |
| RF2506 | CAATCACATCATTAGCAATTTATTCCATG | Amplification of ~200bp *dacS* fragment for qPCR |
| RF2507 | GTTCATCAATATCATCCTTTTCTTTATCC | Amplification of ~200bp *dacS* fragment for qPCR |
| RF2508 | GGATGGGATAGAAGTTTGTAGAAAAG | Amplification of ~200bp *dacR* fragment for qPCR |
| RF2509 | CTCTTCTAATCAGTGATTTCACTCTC | Amplification of ~200bp *dacR* fragment for qPCR |
| RF2510 | CAACATGATTCAGAACAAGATGTTGAG | Amplification of ~200bp *dacJ* fragment for qPCR |
| RF2511 | GCTTGCTTAACTAAATCTTCAACTGC | Amplification of ~200bp *dacJ* fragment for qPCR |
| RF2545 | GGTAAGGAAGCTCTAGAATGTATTG | Amplification of ~200bp *vanR* fragment for qPCR |
| RF2546 | GCAACAACTTCCAAAGGGTTAAATG | Amplification of ~200bp *vanR* fragment for qPCR |
| RF2547 | GCTTTTCGTATGGAATATAAAGCTGC | Amplification of ~200bp *vanS* fragment for qPCR |
| RF2548 | CTTTTCTATTGCCAATAACTCTGGAG | Amplification of ~200bp *vanS* fragment for qPCR |
| RF2549 | GCAGTACTTGAGAATTTGAATACGG | Amplification of ~200bp *vanG* fragment for qPCR |
| RF2550 | GGAATATGATTCTGAGAAACAGCATC | Amplification of ~200bp *vanG* fragment for qPCR |
| RF2551 | CTGTAGATACAAGGTTTCCAAGTATTC | Amplification of ~200bp *vanXY* fragment for qPCR |
| RF2552 | CTGTGATTTGGAAGTGCTACAAAC | Amplification of ~200bp *vanXY* fragment for qPCR |
| RF2553 | CGTATTGCTGTGCCATTCTTTTTTATG | Amplification of ~200bp *vanT* fragment for qPCR |
| RF2554 | CCAATAATTGATGCAGGTAGATACC | Amplification of ~200bp *vanT* fragment for qPCR |
