# Supplementary material for: Identification of pathways to high-level vancomycin resistance in Clostridioides difficile that incur high fitness costs in key pathogenicity traits
Source: PLoS Biol. 2024 Aug 15;22(8):e3002741. doi: 10.1371/journal.pbio.3002741 (PMC11326576; doi:10.1371/journal.pbio.3002741)
Supplement: S3 Table — (DOCX) [file pbio.3002741.s017.docx]

**S3 Table:** Plasmids used in this study

| Plasmid | Characteristics | Source |
| --- | --- | --- |
| ***General Plasmids*** | | |
| pJAK112 | pMTL-SC7215 based vector with added BamHI and SacI restriction sites for cloning. | [3] |
| pJAK143 | *PaLoc* deletion – 1200bp homology arms upstream and downstream of the *PaLoc* for deletion of the entire pathogenicity locus (*tcdD, tcdB, tcdE, tcdA, dxtA)*, except the first codon of *dxtA*. | [4] |
| pJEB002 | *mutSL* deletion – 1200 bp homology arms upstream and downstream of *mutSL* for deletion of the entire *mutSL* locus (*mutS, mutL*), except the first codon of *mutS* and the last 2 codons of *mutL.* | This study |
| pMTL-SC7215 | Allele exchange vector for *codA*-based selection. | [5] |
| ***Plasmids for Barcoding*** | | |
| pJAK081 | pMTL-SC7215 based vector with 1,200 bp homology arms for insertion of DNA sequences between *CD0188* (*pyrE*) and *CD0189* in the *C. difficile* R20291 genome. | This study |
| pJAK201 | *pyrE::*barcode 1 - pJAK081 based vector*,* with a 218 bp insertion containing 9 nt Barcode 1 (AAGTCCTCG). | This study |
| pJAK202 | *pyrE::*barcode 2 - pJAK081 based vector*,* with a 218 bp insertion containing 9 nt Barcode 2 (TCTTGACCG). | This study |
| pJAK203 | *pyrE::*barcode 3 - pJAK081 based vector*,* with a 218 bp insertion containing 9 nt Barcode (AACAACACC). | This study |
| pJAK204 | *pyrE::*barcode 4 - pJAK081 based vector*,* with a 218 bp insertion containing 9 nt Barcode 4 (AACAGGTGG). | This study |
| pJAK205 | *pyrE::*barcode 5 - pJAK081 based vector*,* with a 218 bp insertion containing 9 nt Barcode 5 (ACCGATTAG). | This study |
| pJAK207 | *pyrE::*barcode 7 - pJAK081 based vector*,* with a 218 bp insertion containing 9 nt Barcode 7 (CCTCCAACT). | This study |
| pJAK208 | *pyrE::*barcode 8 - pJAK081 based vector*,* with a 218 bp insertion containing 9 nt Barcode 8 (CGAGGACAT). | This study |
| pJAK209 | *pyrE::*barcode 9 - pJAK081 based vector*,* with a 218 bp insertion containing 9 nt Barcode 9 (CTGGTTCTA). | This study |
| pJAK210 | *pyrE::*barcode 10 - pJAK081 based vector*,* with a 218 bp insertion containing 9 nt Barcode 10 (GGATGTTGG). | This study |
| pJAK211 | *pyrE::*barcode 11 - pJAK081 based vector*,* with a 218 bp insertion containing 9 nt Barcode e 11 (GTCACCAGT). | This study |
| ***Plasmids for Recapitulating Mutations*** | | |
| pJEB019 | *dacS*c*.*548T>C - pJAK112 based vector containing 1,926 bp homology arms centred on a *dacS* 548T>C point mutation. | This study |
| pJEB026 | *dacS*c.714G>T - pJAK112 based vector containing 1,926 bp homology arms centred on a *dacS* 714G>T point mutation. | This study |
| pJEB033 | 1,197,357_1,197,400del – pJAK112 based vector containing 1884 bp homology arms centred on the 1,197,357_1,197,400 deletion. | This study |
| pJEB034 | *vanS*c*.*367_396dup – pJAK112 based vector containing 1942 bp homology arms centred on an intergenic 30 bp insertion in *vanS*. | This study |
| pLMT002 | *dacRS* deletion – pJAK112 based vector containing 975 and 852 bp homology arms up and downstream respectively of *dacRS* for deletion of all except the first codon of *dacR* and last codon of *dacS.* | This study |
| pLMT003 | *dacJ* deletion – pJAK217 based vector containing 975 bp homology arms upstream and downstream of *dacJ* for deletion of all except the first and last codon of *dacJ.* | This study |
| ***qRT-PCR Plasmids*** | | |
| pJEB029 | qPCR 1 - pUC-GW-Kan vector including ~200 bp fragments of *rpoA, dacS, dacR, dacJ* and *rnpA* for qRT-PCR. | This study |
| pJEB032 | qPCR 2 - pUC-GW-Kan vector including ~200 bp fragments of *rpoA, vanR, vanS, vanG, vanXY* and *vanT* for qRT-PCR. | This study |

**References**

3. Fuchs M, Lamm-Schmidt V, Sulzer J, Ponath F, Jenniches L, Kirk JA, et al. An RNA-centric global view of *Clostridioides difficile* reveals broad activity of Hfq in a clinically important gram-positive bacterium. Proc Natl Acad Sci U S A. 2021;118(25). doi: 10.1073/pnas.2103579118. PubMed PMID: 34131082; PubMed Central PMCID: PMCPMC8237595.

4. Ormsby MJ, Vaz F, Kirk JA, Barwinska-Sendra A, Hallam JC, Lanzoni-Mangutchi P, et al. An intact S-layer is advantageous to *Clostridioides difficile* within the host. PLoS Pathog. 2023;19(6):e1011015. Epub 20230629. doi: 10.1371/journal.ppat.1011015. PubMed PMID: 37384772; PubMed Central PMCID: PMCPMC10310040.

5. Cartman ST, Kelly ML, Heeg D, Heap JT, Minton NP. Precise manipulation of the *Clostridium difficile* chromosome reveals a lack of association between the *tcdC* genotype and toxin production. Appl Environ Microbiol. 2012;78(13):4683-90. Epub 20120420. doi: 10.1128/AEM.00249-12. PubMed PMID: 22522680; PubMed Central PMCID: PMCPMC3370502.
