# Supplementary material for: Identification of pathways to high-level vancomycin resistance in Clostridioides difficile that incur high fitness costs in key pathogenicity traits
Source: PLoS Biol. 2024 Aug 15;22(8):e3002741. doi: 10.1371/journal.pbio.3002741 (PMC11326576; doi:10.1371/journal.pbio.3002741)
Supplement: Attachment — Submitted filename: 240509_Response_to_reviewers.docx [file pbio.3002741.s018.docx]

**Academic Editor:**

Having discussed the reviews with the Academic Editor, they agree that the prior work on WalRK increases the need to invest additional effort into follow-up of DacJ and VanT. Just so that nothing gets lost in translation, here's a lightly edited version of the AE's specific advice:

"Rev#2's concern about the novelty of identifying the 2 component signal-transduction system, based on Wal-RK study from David Weiss' group, is valid, as are rev#2 and rev#3's concerns about the advance presented without further functional analyses. The 1st concern should be acknowledged, but reduces the impact of the study, which makes further functional analyses the more important in order to confirm the hypothesized roles of both DacJ and VanT, as well as their role in the fitness defects in the absence of vancomycin. I feel that we should insist that the authors analyse mutant alleles without the influence of possible secondary mutations (i.e. by introducing them in the 'ancestral' background and/or knocking them out/down and overexpressing them to analyse effects on resistance and fitness)."

The *walRK* genes studied by David Weiss's group are unrelated to any of the genes identified in this manuscript. In our response to Reviewer 2 below we have explained that *dacRS* and *walRK* encode different and unrelated two component systems, located on opposite sides of the *C. difficile* genome. Further, the regulated genes are also entirely distinct. To address the concerns about functional data from Reviewer 3 we have substantially expanded our mechanistic analysis of the *dacJRS* system, directly linking DacJ to resistance through its effect on the availability of vancomycin binding sites in the cell wall. We also identified through additional long-read sequencing a previously unknown duplication in *vanS* that results in dramatic upregulation of the genes in the *vanG* cluster and acts synergistically with the *dacJRS*-encoded system to fully recapitulate the resistance seen in our Bc1 evolved isolate.

**Reviewer #1:**

Review Plos Biology
Multiple evolutionary pathways lead to vancomycin resistance in Clostridioides difficile

Clostridioides difficile is an important human pathogen, for which there are very limited treatment options, primarily the glycopeptide antibiotic vancomycin. In recent years vancomycin resistance has emerged as a serious problem in several Gram positive pathogens, but high level resistance has yet to be reported for C. difficile, although it is not known if this is due to constraints upon resistance evolution in this species. The development of vancomycin resistance is investigated by means of experimental evolution. The data in this manuscript shows that high vancomycin resistance leads to considerable fitness costs, both at the level of growth rate, as well as pathogenicity traits.

General scope:

The study is timely, increased antimicrobial resistance of Gram positives is emerging, and there is an increased interest and enough technological advancement to study the development of resistance in surveillance programs.

It is therefore important to investigate what the genetic and population genetic constraints are of the potential development of high-level vancomycin resistance of C. difficile.

Findings:

The authors find alternative resistance mechanisms that lead to high-vancomycin resistance via experimental evolution. Resistance is mediated by either vanT or dacJRS, these seem to be mutually exclusive because these are not found together in evolved end-point isolates. Generally, the high resistance is traded off with considerable fitness effects, both at the level of growth rate, as well as pathogenicity traits that impact transmission.

To my opinion, the findings in this manuscript are sound. I therefore suggest only minor revisions.

Minor:

Line 33: trains = traits

Thank you for catching our typo - corrected.

L33: ' Mutational roadmap to inform genomic surveillance' (last sentence abstract). Although I like this sentence as a sentence, I am not sure whether the outcome of the study warrants this.

This study finds several mutations via experimental evolution that lead to vancomycin resistance, which may all be interesting and relevant to the clinical situation, but unfortunately the relevance of the mutations is not so clear (yet) for the clinical C. difficile isolates. Literature relating to the described mechanisms in the clinical situation would improve the clinical relevance, and also inform better on why genomic surveillance should focus on those particular resistance mechanisms.

I do agree that it would be good for the surveillance community to know about these here described mechanisms, but 'mutation roadmap' based on evolution experiments in a 'not so in vivo like environment' is perhaps overstating the direction that the surveillance should take.

We agree that this could be seen as overstating the importance so have changed the sentence to give a more nuanced view:

*“Moreover, our data identify potential routes to resistance that should be considered in genomic surveillance.”*

Line 81: mutations in the pathway are predicted: not so clear here, predicted by the authors or by literature?

Apologies for the ambiguity. We have changed this to read:

*“Mutations in either pathway are likely to lead to modification of the vancomycin target in the cell wall peptidoglycan and each was associated with defects in growth and sporulation of varying severity.”*

Line 95: 'complete deletion of 18 kb spanning 94 the entire PaLoc that includes the genes encoding both major toxins and associated 95 regulatory proteins, creating strain R20291ΔpaLoc'.

I do understand why the authors delete this region for practical purposes. Do the authors believe that the deletion of this part would have (additional) pleiotropic effects on the developed resistance?

We are not aware of any pleiotropic effects that would impact the development of resistance, although, of course, we cannot completely rule out this possibility. Given the risks associated with generating strains resistant to vancomycin we wanted to ensure that our isolates were entirely avirulent and there are few described mutations sufficient to completely attenuate *C. difficile* virulence. By performing all analysis, including recapitulation of individual mutations, in the same genetic background we sought to minimise confounding impacts and focus on the mutations of interest.

Line 116: Different evolutionary scenarios: Different mutations in the MutSL lineages, faster and higher adaptation. Is this due to more combinatorial power for weak effect mutations, different population genetics leading to the selection of other mutations, or just hitch-hiking?

The genetic data suggest both effects: we observe some mutations in resistance-associated loci exclusively in the hypermutator lines, suggesting an effect of increased supply of beneficial mutations, but variable other loci mutated per line suggesting pervasive hitchhiking of random mutations. We have added a paragraph to the discussion from line 488 to clarify our thinking on this:

“*On average, the* mutSL *hyper-mutating populations became vancomycin resistant more quickly than the equivalent wild-types and this was associated with a dramatically increased accumulation of SNPs throughout the evolution at the population level, and also apparent in the endpoint evolved clones. Evidence from both population and endpoint sequencing suggests that, at least in part, this reflects the hyper-mutators exploring a wider array of possible beneficial mutations. However, the wide variation in the both numbers and nature of accompanying mutations suggests that many of these may also be random hitchhiking mutations*.”

Line 123: Significant variation in MIC within evolved populations. Do the authors have an idea how the less resistant mutants can stay in the more highly-resistant population? And how important this finding may be for the clinical situation?

In nearly all cases the observed difference in MIC was only 2-fold and given the granularity of these measurements, the reality could be less than 2-fold. There are many possible explanations for the variability, including the different environmental conditions (broth vs agar, media composition, cell density, etc.), epigenetic or regulatory effects (e.g. *C. difficile* has phase variable c-di-GMP phosphodiesterases that could have broad impacts on cell physiology), or even the phenomenon of heteroresistance. We have avoided speculation in the manuscript without further data.

Line 156: Indels and copy-number variation missing in the analysis of the vancomycin resistant mutants? Line 183, does mention deletion though. Unclear how important indels and copy-number variants are for the development of resistance.

We did not observe any copy-number variants in any of our datasets, including in our newly added long-read sequencing of the five wild type endpoint clones. In the main text figures for clarity we have chosen to focus on non-synonymous mutations, including indels and SNPs. A full list of these, and also including intergenic and synonymous mutations, is included in S2_Data for the individual endpoint isolates and in S3_Data for the populations. As the vast majority of within-gene indels identified resulted in frameshifts, we have referred to them as such in text - 31% of identified mutations were frameshifts as a result of single nucleotide indels. Interestingly, long read resequencing of the endpoint isolates Bc1-5 did identify two additional substantial indels in Bc1, a 44 bp intergenic deletion and a 30 bp duplication in the *vanS* gene. Analysis of these mutations is described in the manuscript from Line 344 onwards and in the associated new figures and table.

Line 200: Evolution experiments and assays are performed under rich medium conditions. Do the authors believe that the pleiotropic effects (that potentially limit the clinical importance of the found resistance mechanisms) also affect pathogenicity traits in other settings. In other words, could it be that the fitness costs associated with resistance development, on both the level of growth and pathogenicity, would be less (or more?) severe under other conditions?

Having said this, resistance is also tested on rich media in the clinic (in which the authors show that there is this pleiotropic cost), perhaps this also leads to an under-detection of vancomycin resistance in C.difficile (as the authors mention, there is quite some MIC differences in an evolved population, and more resistant mutants have a higher cost)?

To be clear: I am not asking for more experiments.

Thank you for not asking for evidence for this as it’s a huge, if fascinating, question! In general, we suspect it is likely that nutrient poor / *in vivo* environments would further exacerbate growth defects. In future we plan to assess fitness in an *in vitro* gut model seeded with natural human microbiota. However, those experiments are extremely time consuming and expensive so will form part of a follow-on project. We also hope to extend our analysis to clinical isolates more broadly so that we can address the latter.

Line 228: possible that affect regulation.. how?

This intergenic mutation could impact a regulatory sequence, such as a transcription factor or nucleoid associated protein binding site, associated with the *glsA* promoter. Unfortunately, it’s impossible to predict this in *C. difficile*, even for regulators with large regulons, so we felt it important to express the uncertainty. We have changed the text at Line 234 to clarify this:

*“...but it is possible that they affect regulation of* glsA *through alteration of the binding site for a regulatory protein or RNA”*

Line 270: How many fold upregulation? 4.8-94.6

This was our attempt to express the range as the magnitude of difference varies considerably across the three genes and two mutations but this could certainly have been clearer. To clarify we have changed this to:

*“Either point mutation resulted in a substantial upregulation of expression of all three genes, varying from 4.8 fold increased transcription of dacS in R20291ΔPaLoc dacSc.714G>T to 94.6 fold increased transcription of dacJ in R20291ΔPaLoc dacSc.548T>C. Interestingly, these effects were independent of vancomycin suggesting that the two-component system responds to an as yet unknown signal.”*

Line 272: It is unclear to me how the overexpression of DacJ leads to the reduction in vancomycin binding sites. Could the authors explain?

Apologies, that could have been clearer. We have added new data to directly demonstrate that DacJ activity reduces the availability of vancomycin binding sites in the cell wall - see new Fig. 4. *dacS* mutations that increase *dacJ* expression decrease fluorescent vancomycin labelling and deletion of *dacJ* in a resistant clone partially recovers this labelling. This new data is described in an extensively rewritten section that starts at line 313:

*“We hypothesised that the overexpression of DacJ, and the resulting removal of terminal D-Ala residues from peptidoglycan precursors, depleted vancomycin binding sites within the cell wall resulting in the observed reduction in vancomycin susceptibility in both strains with mutated dacS.”*

Line 351: Delayed emergence of vanT variants - relate back to potential importance of co-occurrence with ComR?

We left this a little vague as there isn’t a consistent secondary mutation or class of mutation that preceded all *vanT* variants. We have also struggled to reproduce VanT-dependent resistance by recapitulating individual mutations in a clean genetic background - we have explained this in more detail in our response to Reviewer 3 below.

Lines 499 and onward; analysis also included CNV and indels?

No CNVs were detected in any of our datasets. Many indels resulting in frameshifts were detected (31% of all within gene variants) and these are detailed in the supplemental data files with those affecting protein sequence also included in the in-text figures. Subsequent long read sequencing of the five R20291Δ*PaLoc* evolved endpoint clones revealed two larger indels in Bc1 (a 44 bp intergenic deletion and a 30 bp duplication in *vanS*) that had been missed by our high-throughput illumina pipeline. We have included that new data in this revision and this is described from line 344 onwards, Table 1 and Fig 5.

**Reviewer #2:**

General Comments:

The manuscript by Buddle et al describes the development of vancomycin resistance in C. difficile during growth under antibiotic pressure. The authors demonstrate that although high level resistance is not common among clinical isolates, significant vancomycin resistance can be achieved under selection, although with fitness costs. They explore a series of mutations that are suspected to contribute to target changes within peptidoglycan that reduce the impact of vancomycin on cell wall growth. The identification of potential mutations that confer resistance to vancomycin are of great importance, given that this is the primary antibiotic therapy for CDI. However, the locus the authors expand on as previously unidentified, were characterized in a 2022 paper, reducing the novelty of the findings. Further, the manuscript does not determine the nature of these or other mutations identified in the study to give insight into the specific mechanisms that lead to increased resistance. Additional exploration of the findings could provide helpful insight into the potential development of resistance in this pathogen.

Major comments:

A major finding reported is the identification of a genetic locus that the authors refer to as dac genes. Although the authors state that "these genes had not been previously implicated in vancomycin resistance", there appears to be a publication describing that (PMC9210968 JBac 2022). This is the previously characterized WalRK system.

The previously characterised *walRK* genes are unrelated to the *dacRS* system described here. The strain R20291 orthologues of *walR* (CD630_17820, CDR20291_1677) and *walK* (CD630_17830, CDR20291_1678) are on the opposite side of the genome from *dacRS* (CDR20291_3437 and 3438). Both operons encode two component systems, however WalR displays only 28% amino acid identity with DacR and WalK shares only 20% with DacS. These values are consistent with any pair of unrelated response regulators or histidine kinases.

Throughout the manuscript the authors report mutations in genes, but they do not provide follow up to determine the nature of the mutations, or independently delete the genes, which limits the utility of the work. It is not clear if transcription or function are disrupted in most cases, GOF, LOF, etc. However, the authors make conjectural statements based on presumed functions of the mutations, though multiple mutations are present throughout the genome.  (For example, at Ln 231-234, they mention a substitution in CD3034, suggest its function in D-aa synthesis, and draw conclusions within the results regarding the importance of vanT to resistance.)

The exact nature of each mutation identified is detailed in supplemental data files S2 and S3. In the text we have focused on non-synonymous mutations as these are most likely to alter the function of the encoded protein. These would not normally be expected to impact transcription.

There are 114 individual mutations in the 10 resistant endpoint isolates. Recapitulating this many is impractical given the technical challenges of genetic manipulation in *C. difficile* so we have chosen to focus on those predicted to have the greatest impact on resistance and those that give insight into novel biology. Where we do not have data on the consequences of an individual mutation we have avoided speculating as to GOF vs LOF, etc. However, we do not believe it is inappropriate to suggest the possible role of a protein based on the gene annotation as in the case of CD3034.

To support our conclusions on the contribution of the *dacJRS*-encoded system to vancomycin resistance we have added substantially more data, including analysis of a *dacRS* deletion strain, single, double and triple mutants in *dacS*, *vanS* and *R20291_0979*, that fully recapitulate the mutations found in the Bc1 evolved endpoint clone, and a *dacJ* deletion in the endpoint clone Bc1. This new data is described in an extensively rewritten section that starts at Line 289 and included in an altered Figure 3 and in new Figures 4 and 5 and supplemental Figures 7-9.

The authors reference "CD" numbers as locus tags. CD is the prefix for the strain 630, but these locus tags do not match that reference genome and it is not the strain the authors use. It is most likely they mean to refer to the CDR20291_ locus tags. Please clarify and amend throughout the manuscript. Also, locus tag numbers should be provided in addition to any gene names to ensure clarity.

All genes are identified using the formal CDR20291_XXXX locus tag identifiers in the spreadsheets listing all of the mutations we describe. We opted to use the shorter CD in text to improve readability. We have clarified this choice on first usage in text:

*“In the 5 wild-type vancomycin selected lines we observed parallel evolution occurring at three genomic loci:* vanT *in 3 clones,* CDR20291_3437 *(simplified to* CD3437 *hereafter) in 2 clones and* comR *in 2 clones.”*

We have opted not to make this change in the figures as it would make the text very hard to read.

MIC methods should state the number of cells used as inoculum, rather than optical density with volume, which can vary by medium and strain.

For all engineered strains described in the paper, OD_600nm_ displayed a good correlation with CFU/ml. At the optical density used to normalise MIC assays, total viable counts vary from a minimum of 1.06 x 10^7^ to a maximum of 1.83 x 10^7^. We have added these details to the methods:

“*MICs were obtained via standard agar dilution methods [50]. Briefly, overnight cultures were adjusted to OD_600nm_ 0.1, corresponding to between 1.06 x 10^7^ and 1.83 x 10^7^ CFUs for all strains analysed, and 2.5 µL samples were spotted in biological triplicate and technical duplicate onto BHI plates with ranging antibiotic concentrations.*”

Minor comments:

Growth curves should be plotted on log scale and measurements of growth cannot be negative OD values.

Although it has long been convention to plot growth on a log scale to allow visual comparison and measurement of growth rate from slope, it often (as here) makes it hard for the reader to see the differences between strains. As the GrowthCurver R package we have used can calculate growth parameters directly from the raw data, we chose the most easily interpretable format for the presented graphs.

These growth curves were performed anaerobically in parallel in 96 well plates in a Cerillo Stratus plate reader. Slight variation in optical measurement across the plate is not uncommon and can result in small negative values in some wells at the start of the experiment. Our analysis involved 8 replicates of each strain and we have not excluded any negative data points in the early time points.

Sporulation assays- According to the literature, 65C is not sufficient to consistently kill C. difficile vegetative cells. Controls that cannot sporulate are necessary to confirm spore counts.

This is a fairly standard sporulation assay that we have published many times before and which we have shown to be robust and highly reproducible (e.g. Dembek et al. 2015. MBio e02383-14; Kirk et al. 2017. Sci Trans Med eaah6813; Alabdali et al. 2021. Anaerobe 70:102379; Galley et al. 2024. JBC 300:105529). The effectiveness of this, and less harsh treatments, to kill vegetative cells has also been demonstrated previously by many other groups, for example:

Donnelly et al. 2017. MBio 8:e02085-16 - 60-65°C for 25 min.

Fimlaid et al. 2015. PLoS Genetics 9:e1003660 - 60-65°C for 25 min.

Goulding et al. 2009. Infect. Immun. 77:5478-5485 - 56°C for 20 min

Permpoonpattana et al. 2001. J Bact 193:6461-6470 - 60°C for 20 min.

The effectiveness is also demonstrated in each of the sporulation assays shown in supplemental figures 2, 8 and 9. At the first time point in all assays the heat treatment reduced viable CFUs by >6 logs, with the surviving CFUs being entirely consistent with a small number of spores being present even in actively growing R20291 cultures.

Ln 46- This is a global issue; please restate and provide additional references

While we agree that these issues are global, we also feel that one of the largest health systems in the world recommending vancomycin as the front-line antibiotic is worthy of note. To emphasise the importance and ubiquity of these issues we have revised the sentence to read:

*“Due to increasing incidence of resistance and consequently poor patient outcomes, use of metronidazole has declined in recent years as a result in changing clinical guidelines [7, 8]; vancomycin is now the recommended front line antibiotic in the UK [9] and has been widely adopted worldwide [10].”*

Ln 60-62- Please provide a reference to support the statement about vanG

The relevant sources are cited at the end of the next sentence but this was a little awkwardly constructed. For clarity we have changed this to:

*“However, increased expression of the* vanG *cluster does appear to be associated with reduced susceptibility to vancomycin; mutations in the* vanSR*-encoded two component system, reducing vancomycin susceptibility through derepression of the* vanG *cluster, have been reported in both clinical isolates and laboratory evolution experiments [19, 20].”*

Ln 139- Loadings is not a universally used term and its meaning here is not clear

‘Loadings’ is a standard term in principal component analysis that is used to describe the contribution of an original variable to the principal component. In this case the Loadings were sporulation efficiency, growth, MIC, cell length.

Ln 188- It is relevant to note that the gene referred to as CD3124 encodes a predicted VanS-like kinase

This gene is not annotated as such in the majority of *C. difficile* genomes that we have examined, including the R20291 genome from the Wellcome Trust Sanger Institute, and in most cases is annotated as HAMP domain-containing sensor histidine kinase. There does not appear to be any explanation for why this annotation was applied in a small number of genome annotations - in many cases annotation is largely automatic with little to no curation so determining reasoning is almost impossible. Indeed R20291_3124 only shares 25% identity with the *C. difficile* VanS.

Fig S2- a wildtype control is needed for reference

The ancestral strain for all of the described mutants is R20291Δ*PaLoc* and that is included as the comparator for each assay. We believe that this is the correct control strain as we are assessing the impact of the additional mutations and not the Δ*PaLoc* mutation which is present in all of the strains.

Strain Fitness analysis- Methods state that cells were inoculated, incubated for 1 hr at 37C, then measured every 3 minutes for 24. Why would cells be preincubated? Why are only 8 hours shown on graphs.

We have found that a one hour preincubation before placing the 96 well plate into the plate reader serves to reduce apparent edge effects in measurements. We originally based this on a publication that developed this technique for cell culture-based applications (DOI: 10.1177/1087057103256465) but we found this to be effective for *C. difficile* as well. We have only presented the first 8 hours to be consistent as the wild type controls are in stationary phase at that point.

Fig S2- 1) Days should not be shown as "T0" etc. T traditionally refers to the timepoint of sporulation  2) BC2 and BC3 have different y-axis demarcations than the other graphs
3) What timepoint is statistically assessed for the reported p values?

1) We used T0 in the more general sense as the first timepoint but to avoid ambiguous overlap with alternative definitions specific to sporulation we have removed the Ts and rephrased the legend.

2) Thank you for catching this embarrassing error. We had overlooked that 10^1^-10^9^ was displayed for those two graphs rather than the 10^-1^-10^9^ range for all of the others. This has now been corrected.

3) As stated in the figure legend the statistical analysis is based on the total area under the curve, not a single timepoint, for each strain compared using Dunnett's T3 multiple comparisons test. We used area under the curve to provide a more holistic overview of sporulation efficiency as it combines both the rate of sporulation and the total number of spores produced in a single measure.

Fig S3- How many biological replicates were performed for these assessments?

This phase contrast microscopy was performed in biological triplicate, with images from all three experiments pooled for cell size analysis. The legend has been amended to include these details.

Fig S4- 1) Please provide subfigure letter designations. 2) the data vis showing both SNP frequency and MIC as several overlapping lines, colors, and shading is difficult to interpret

1) We have added panel labels to Figure S4 and also to main text figure 2 for consistency and changed the legends to reflect this.
2) We appreciate that these data are hard to interpret, this is the reason, at least in part, why we have largely focused the narrative in the paper on the variants observed in the 5 wild type populations. This figure, showing essentially the same information as both a circle plot and line graph, supported by the full numerical data in the S3 Data supplemental spreadsheet, is our best effort to support the reader’s understanding. We also included the fixation data in S4 to help highlight the most important mutations from out of the complex dataset.

Fig S6- legend states that mutations occurring within genes were coloured according to their barcoded replicate line, but the bars are all blue. What are barcoded replicate lines?

In this context ‘coloured’ refers to the method by which pathways are mapped using the tool KEGG Colour Mapper and not to how the data is displayed. This is rather confusing, so we have rephrased the legend to read:

*“Genes impacted by mutations during evolution (except those in the transiently hyper-mutating Bc1 P20) were visualised in KEGG (Kyoto Encyclopedia of Genes and Genomes) colour mapper and assigned to cellular pathways. Two-component systems and ABC transporters were the best-represented functional classes.”*

**Reviewer #3:**

In this manuscript, Buddle and colleagues performed in vitro evolution of Clostridioides difficile under increasing vancomycin concentrations paired with genome sequencing to investigate the genetic basis of evolved vancomycin resistance in C. difficile. Among other mutations, the authors identified mutations in the VanG-type cluster, particularly in vanT, as well as an additional operon that the authors have termed dacJRS. While mutations in vanR and vanS have been previously implicated in vancomycin resistance in C. difficile, the authors find that vanT mutations are more common in their in vitro evolution system and do not currently have a known role in vancomycin resistance. In addition, dacJ encodes a putative D,D-carboxypeptidase, a peptidoglycan-modifying enzyme known to confer vancomycin resistance in other bacteria. By recapitulating dacS mutations, the authors find that DacS affects expression of dacJ and propose that DacJ confers resistance to vancomycin in C.
difficile. Importantly, most vancomycin-resistant isolates from their evolution experiment had growth and sporulation defects, suggesting that fitness trade offs may limit the evolution of vancomycin resistance in clinical strains. I am quite enthusiastic about this work, as it has important implications for our understanding of antibiotic resistance mechanisms in C. difficile and sheds light on the potential evolutionary tradeoffs this pathogen faces. However, additional experiments are needed to support and strengthen the authors' proposed model and more is needed to define the role of vanT and dacJ in vancomycin resistance in C. difficile.

1. Authors suggest DacJ is an important resistance mechanism to vancomycin in C. difficile, which is based on data from dacS-mutated strains showing increased dacJ expression. However, the role of DacJ is not directly tested. Overexpression of dacJ in the wildtype strain or knockout/knockdown dacJ in the dacS-mutated background would determine whether the increased vancomycin resistance observed in dacS mutants is due to increased dacJ expression or a distinct mechanism.

We agree that the evidence for involvement of DacJ was largely circumstantial. To firm this up we have deleted *dacJ* in the Bc1 resistant endpoint clone, which reduced the vancomycin MIC by 8 fold. We have also shown that *dacJ* expression is not affected by a *dacRS* deletion, indicating that the two-component system is a positive regulator of the system. These data have been added to a new Figure 4, table 1 and an expanded Figure 3.

2. The role of DacJ as a D,D-carboxypeptidase has not been validated in C. difficile, thus these experiments would be needed to conclude that DacJ is capable of conferring vancomycin resistance in this pathogen.

We have been unable to directly validate the enzymatic activity of DacJ *in vitro* as the enzyme is lethal when expressed in *E. coli*. This is likely due to the predominance of 3-4 crosslinks in the *E. coli* cell wall, formation of which could be inhibited by D,D-carboxypeptidase activity trimming Lipid II precursors prior to flipping. Instead, we compared labelling of WT and resistant strains with fluorescent vancomycin-bodipy. In the WT we observed mid-cell labelling as expected, indicating the presence of D-Ala-D-Ala containing peptidoglycan precursors at the site of maximal PG growth. By contrast, *dacS* mutants which overexpress DacJ show no labelling, directly connecting expression of this enzyme to the availability of vancomycin binding sites. The Bc1 endpoint clone also lacked detectable vancomycin-bodipy labelling at the mid-cell but labelling was partially restored in a *dacJ* mutant. This partial restoration is consistent with the intermediate vancomycin MIC we observed for the Bc1Δ*dacJ* strain and the presence of a parallel *vanG*-based resistance mechanism in the Bc1 background (see below). We have included this data, along with quantification of vancomycin-bodipy labelling at mid-plane of individual cells from each strain, in the new Fig. 4.

Given that the *dacS*c.714G>T SNP only partially reproduced the vancomycin resistance seen in Bc1, we re-sequenced Bc1-5 by nanopore and polished using the illumina data. We repeated the SNP calling with the new high-quality closed genomes, which largely confirmed our earlier analysis. However, two large indels were identified in Bc1, a 30 bp duplication within the ORF of *vanS*, encoding the *vanG* cluster histidine kinase, and a 44 bp deletion in a very small ORF annotated as *R20291_0979*. However, we believe that the latter is a misannotation and that this deletion is in an intergenic region. We have added these mutations to our analysis with *dacS*c.714G>T in all combinations of single, double and triple mutations. The full MIC data is included in table 1 but the headline is that the *vanS* and *dacS* double mutant and the triple mutant fully reproduce the MIC of Bc1. We suspected that the *vanS* insertion was impacting expression of the rest of the *vanG* cluster and we have confirmed this by qPCR. These data have been included in the new Figure 5.

3. Mutations in vanT are proposed as path to vancomycin resistance; however, the authors do not validate the role of vanT in C. difficile. Recapitulating vanT mutations and testing vancomycin resistance would increase the impact of this work by further clarifying the role of the C. difficile VanG cluster in vancomycin resistance.

We have attempted to recapitulate the *vanT* mechanism in a clean genetic background but with no success to date. We have generated strains with the *maa* and *vanT* SNPs from Bc5 (singles and double), the *comR* and *vanT* SNPs (singles and doubles) from both Bc3 and Bc4 and the *vanT* mutation from Bc11. Of these 7 mutant strains, the only increases in MIC observed were associated with the Bc4 *comR* SNP - a 4-fold increase - and the Bc3 *comR* SNP - a 2-fold increase. Nanopore sequencing revealed no additional mutations in the Bc3-5 evolved endpoint clones. Parallel evolution of these loci and previous work on the *vanG* cluster makes a very strong case for the involvement of *vanT* in resistance. However, our inability to recapitulate the phenotype suggests that there is a further non-heritable mechanism at play. We are investigating possible epigenetic mechanisms but this will take a long time to unravel, if indeed we can figure it out at all.

We have referred to our inability to recapitulate the *vanT*-associated phenotype in the discussion from line 439.

*“Unfortunately, we have been unsuccessful in recapitulating vanT-mediated resistance, despite introducing all 4 of the identified vanT mutations individually into the parental R20291ΔPaLoc strain. Indeed, recapitulation of all SNPs and indels identified in the Bc3 endpoint clone in R20291ΔPaLoc resulted in only a modest 2-fold increase in vancomycin MIC. Given the phenotypic differences between apparently genetically identical strains we hypothesise that currently unknown epigenetic or regulatory effects may play an important role in this mechanism of resistance.”*

4. A main and perhaps most intriguing finding of this work is that the evolution of vancomycin resistance results in fitness costs which may curb widespread emergence of vancomycin-resistant C. difficile. However, it is unclear whether the observed growth and sporulation defects are a direct result of specific vancomycin resistance mutations or a cumulative effect of multiple non-specific mutations. This is particularly a concern due to the fact that the hypermutator isolates had more severe fitness defects than the isolates from the wildtype background. Growth rate and sporulation of the recapitulated dacS-mutated strains should be tested to determine whether growth and sporulation defects are a direct result of this resistance pathway.

We have assessed growth of the recapitulated *dacS*c.714G>T strain (copying Bc1) as well as in the *vanS*, *dacS*+*vanS*, *vanS*+*0979* and *dacS*+*vanS*+*0979* mutant strains and also analysed sporulation efficiency of the triple mutant. In general, all strains with the *dacS* mutation very closely replicated the growth defects seen in the evolved Bc1 endpoint clone, suggesting that this mutation is responsible for the growth defect in Bc1. The Bc1 clone sporulated normally and this was also seen with the recapitulated triple mutant as well. The Bc8 and Bc9 endpoint clones (both *dacS*c.548T>C) had small growth defects and also displayed reduced sporulation. Recapitulation of this *dacS* allele did not impact sporulation, suggesting that the other mutations seen in the hypermutating Bc8/9 were responsible for the observed sporulation phenotypes. Interestingly, however, *dacS*c.548T>C resulted in a clear growth defect similar to that seen in Bc1 and *dacS*c.714G>T and more severe than the minor effect seen in the Bc8 and Bc9 endpoint clones. This suggests that additional mutations in the hypermutating lineages are compensating for *dacS* and serving to increase fitness. This apparent evidence in favour of refining mutations is in line with experimental evolution theory.

The additional growth and sporulation analysis data is reported in three new supplemental figures - S7-S9 Figs.

5. Unless the mutant is nonviable, the authors should also consider doing the same growth and sporulation assays in a vanT mutant, as described above.

We have analysed the impacts of the individual mutations identified in the Bc4 endpoint clone, a strain that displayed both growth and sporulation defects. The *comR* mutation on its own resulted in a severe growth defect and also delayed sporulation slightly. The *vanT* mutation on its own actually enhanced growth (higher stationary phase OD) but had no effect on sporulation. Combination of *comR* and *vanT* mirrored the effects seen in the *comR* single mutant. As we have not been able to replicate vancomycin resistance with these strains we have not included the growth and sporulation data in the revised manuscript but these are included below for review purposes.

**Growth and sporulation of Bc4-recapitulated strains. A** Growth over time in rich media (TY broth) was measured at 600 nm in a 96 well microplate spectrometer. Growth of R20291Δ*PaLoc* *comR*c.1337G>T (*comR**), R20291Δ*PaLoc* *vanT*c.271C>A (*vanT**), and R20291Δ*PaLoc* *comR*c.1337G>T *vanT*c.271C>A (*comR** *vanT**) (coloured lines) was compared to R20291Δ*PaLoc* (black lines)*.* Growth of endpoint clone Bc4 (coloured line) and its matched control (black line) from S1 Fig was included for comparison purposes. Shown are the mean and standard deviation of repeats, assayed at minimum in biological and technical triplicate. For each strain, area under the curve was determined using the GrowthCurver R package and these were compared using Student’s t-tests with Welch's correction, with the P value shown on each graph. **B** Sporulation efficiencies of R20291Δ*PaLoc* *comR*c.1337G>T (*comR**), R20291Δ*PaLoc* *vanT*c.271C>A (*vanT**), R20291Δ*PaLoc* *comR*c.1337G>T *vanT*c.271C>A (*comR** *vanT**) and endpoint clone Bc4 (coloured lines) compared to the parental R20291Δ*PaLoc* (black lines). Stationary phase cultures were incubated anaerobically for 5 days with samples taken daily to enumerate total colony forming units (CFUs, dotted lines) and spores (solid lines), following incubation at 65°C for 30 min to kill vegetative cells. Shown are the mean and standard deviations of biological triplicate assayed in triplicate. For each strain, spore CFU area under the curve was determined using Graphpad Prism and these were compared using Dunnett's T3 multiple comparisons test with the adjusted P value shown on each graph. * = significant difference, N.S. = not significant.
